# A Rare Case of Spontaneous Hemorrhage in a Giant Accessory Spleen in a Child

**DOI:** 10.1155/2019/1597527

**Published:** 2019-01-20

**Authors:** Gianpaolo Grilli, Valentina Pastore, Vincenzo Bertozzi, Annarita Nunzia Cintoli, Francesco Perfetto, Maria Nobili, Francesco Canale, Luca Macarini

**Affiliations:** ^1^Radiology Unit, Riuniti Hospital, Foggia, Italy; ^2^Radiology Unit, University of Foggia, Foggia, Italy; ^3^Pediatric Surgery Unit, Riuniti Hospital, Foggia, Italy

## Abstract

Accessory spleen (AS) is a condition found in about 20% of patients and is most commonly located in the hilar region of the spleen. It is more often asymptomatic, single, and smaller than 2 cm. In the present study, we report the rare case of a 13-year-old boy with giant accessory spleen underwent spontaneous intrasplenic hemorrhage who presented with recurrent abdominal pain. Contrast-enhanced MRI was mandatory for the diagnosis before surgical procedure.

## 1. Introduction

An accessory spleen (AS) or splenunculus is a congenital anomaly consisting of the ectopic normal splenic tissue separated from the main body of the spleen [1] and most commonly located in the hilar region of the spleen (75%), along the mesentery or splenic vessels [2], in the greater omentum [3] or in the tail of the pancreas (1-2%) [4]. AS is seen in approximately 10% to 15% of individuals [1, 2] and is caused by the failure of some primordial buds of the splenic tissue to fuse in the dorsal mesogastrium during the 5th week of embryonic organogenesis [5] or to an extreme degree of splenic lobulation with pinching off of the splenic tissue [6]. ASs are usually single, smaller than 2 cm, and incidentally founded in asymptomatic patients [7]. Sometimes, AS can mimic lymphadenopathy or tumors in other organs and in hematological disorders that can take over the function of the original spleen after splenectomy. In rare cases, AS can cause pain because of acute pedicle torsion (which is most often seen in children), recurrent abdominal pain, cysts, abscesses, hemorrhage, or spontaneous rupture.

## 2. Case Report

**Figure 1:**
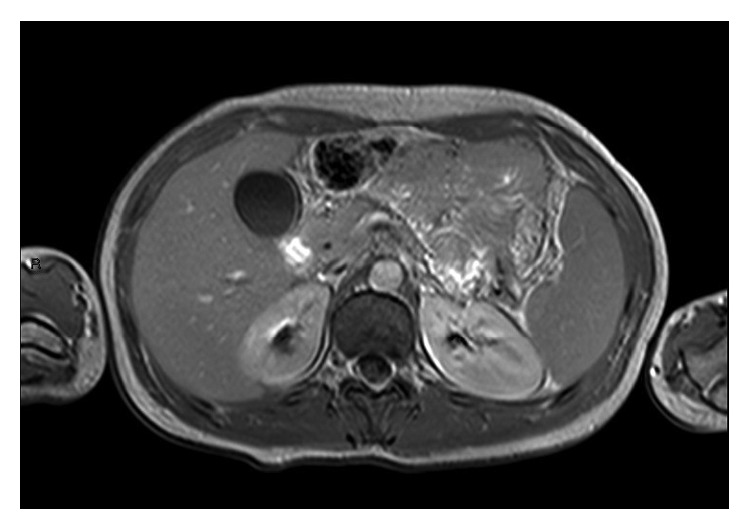
MRI showing normal spleen.

**Figure 2:**
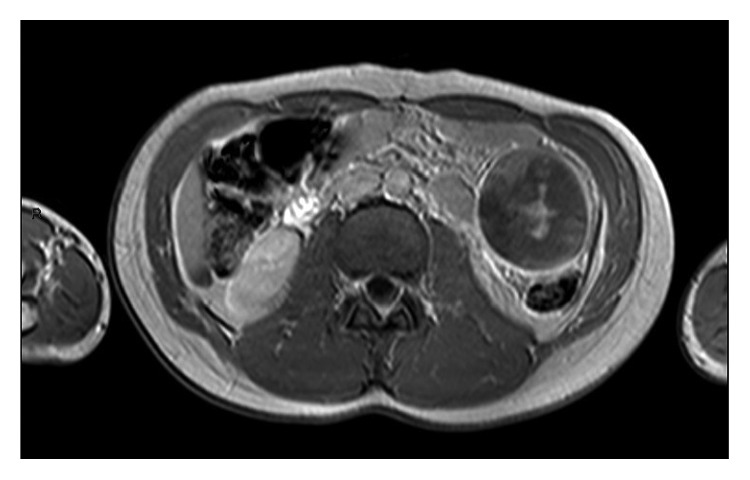
T1-weighted gadolinium-enhanced axial view of MRI showing hemorrhage within the AS.

**Figure 3:**
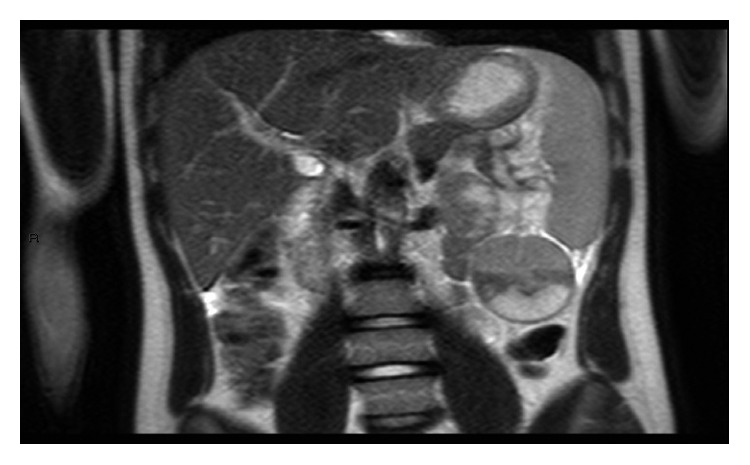
T2-weighted gadolinium-enhanced coronal view of MRI showing hemorrhage within the AS.

A 13-year-old boy presented to pediatric surgery unit due to abdominal pain in the left abdomen without fever, nausea, vomiting, or genitourinary symptoms. He had no history of trauma or injury or operative treatment. His family history revealed no significant disease. On admission, the patient presented blood pressure 120/80 mmHg and 80 bpm, and all laboratory data were within normal limits. Examination of the abdomen showed pain in the left abdomen but negative McBurney or Bloomberg signs. Plain Rx of the abdomen did not show signs of intestinal obstruction or perforation. Abdominal US showed a mass 7 cm in diameter, located anteriorly and inferiorly to the spleen with similar structure. Abdominal MRI with contrast confirmed previous finding and, in addition, showed an area of intracapsular hemorrhage in the mass (Figures 1–3). Diagnosis was intracapsular hemorrhage in a giant accessory spleen. Then, the child was brought to OR. Removal of the accessory spleen was made by left subcostal incision without difficulties. The child resumed liquid diet the day after and was discharged on the 3rd postoperative day. The histological examination confirmed the diagnosis.

## 3. Discussion

An accessory spleen, in other words supernumerary spleens, splenunculi, or splenules, is a congenitally duplicated splenic tissue that is separated from the main body of the spleen and is observed in about 10%–30% of patients in autopsy series and 16% of patients undergoing contrast-enhanced abdominal CT [8]. The most common location is splenic hilum and pancreatic tail (25%), but AS can occur anywhere in the abdomen and even in the pelvis and scrotum. AS usually measures 1 cm in diameter and is single, but its size and number can vary [9]. AS usually is incidentally detected and asymptomatic but can be of clinical importance in malignancy patients in whom it could be misinterpreted as metastatic lymph node, in patients with splenic trauma in whom it could be clinically important to preserve splenic tissue in case of splenectomy and also in case of hypersplenism to avoid recurrent disease. Sometimes, AS can cause pain because of acute pedicle torsion (which is most often seen in children), recurrent abdominal pain, cysts, abscesses, hemorrhage, or spontaneous rupture. So, a noninvasively characterization of the lesion is necessary by using US, CT, MRI, and Tc-99 m sulfur colloid scintigraphy. On an US, a typical AS is shown as a well-defined lesion, and administration of an intravenous contrast agent is helpful for the visualization of the vascular hilum [10]. On an unenhanced CT scan, a typical AS is depicted as a well-marginated mass while on a contrast-enhanced CT scan, the lesion is homogenously enhanced as splenic parenchyma [11] and shows a feeding artery from the splenic artery. MRI using superparamagnetic iron oxide as a negative contrast is also helpful for the detection of an AS [12], and in both T1-weighted and T2-weighted images, the reduction of the signal intensity appears while on high *b*-value DWI accessory, the splenic tissue shows marked hyperintensity [13]. Scintigraphy with Tc-99 m phytate may be the most useful method to evaluate a functional AS but sometimes it is difficult to perform [14]. However, the definitive diagnosis is determined by a histopathological examination of the surgical specimen. AS generally does not require therapy which becomes mandatory when AS is involved with hematologic or systemic disorders or when becomes symptomatic [15]. Treatment options can range from conservative approach (in case of asymptomatic AS) to surgical management. Also vascular embolization, when an independent vascular pedicle is found, can be used [16]. The choice for surgical approach is surely influenced by the attitude of the operating surgeon toward minimally invasive techniques such as laparoscopy or robotic surgery (which are nowadays considered as the gold standard in uncomplicated cases) or open technique [17]. In our patient, recurrent abdominal pain was probably due to short lasting ischemia caused by intermittent torsion-detorsion which was possible for the incomplete fixation of the spleen to the gastrosplenic and splenorenal ligaments and for a longer vascular pedicle [18]. The operating surgeon had the feeling to go for an open approach because of the risk of ruptured AS and associated hemorrhage. However, at the present time, most of the pediatric surgeons approach this disease by laparoscopy or robotic surgery in elective cases. To our knowledge, there are no reports in the literature about recurrent abdominal pain in children due to giant AS, and spontaneous intrasplenic hemorrhage is, furthermore, an extremely rare complication. In conclusion, on the basis of these observations, the authors think it is important to recognize that the giant AS can be a rare cause of recurrent abdominal pain in children, and an awareness of an AS and familiarity with typical imaging findings are necessary to make a precise preoperative diagnosis.
